# The Cyanthin Diterpenoid and Sesterterpene Constituents of *Hericium erinaceus* Mycelium Ameliorate Alzheimer’s Disease-Related Pathologies in APP/PS1 Transgenic Mice

**DOI:** 10.3390/ijms19020598

**Published:** 2018-02-17

**Authors:** Tsai-Teng Tzeng, Chien-Chih Chen, Chin-Chu Chen, Huey-Jen Tsay, Li-Ya Lee, Wan-Ping Chen, Chien-Chang Shen, Young-Ji Shiao

**Affiliations:** 1 Institute of Biopharmaceutical Science, National Yang-Ming University, Taipei 112, Taiwan; fly23242530@hotmail.com; 2 Department of Cosmetic Science, Chang Gung University of Science and Technology, Kweishan, Taoyuan 333, Taiwan; chen37426972@gmail.com; 3 Biotechnology Center, Grape King Bio Ltd. Chung-Li, Taoyuan 320, Taiwan; gkbioeng@grapeking.com.tw (C.-C.C.); ly.lee@grapeking.com.tw (L.-Y.L.); wp.chen@grapeking.com.tw (W.-P.C.); 4 Institute of Neuroscience, Brain Research Center, School of Life Science, National Yang-Ming University, Taipei 112, Taiwan; hjtsay@ym.edu.tw; 5 National Research Institute of Chinese Medicine, Ministry of Health and Welfare, Taipei 112, Taiwan; yshiao@nricm.edu.tw

**Keywords:** Alzheimer’s disease, APPswe/PS1dE9 transgenic mice, erinacines, amyloid plaque, astrocytes, microglia, neurogenesis

## Abstract

*Hericium erinaceus* was used in traditional Chinese medicine for physiologically beneficial medicines. Recently, it has become a candidate in causing positive brain health-related activities. We previously reported that *Hericium erinaceus* mycelium ameliorates Alzheimer’s disease (AD)-related pathologies. To reveal the role of the cyanthin diterpenoid and sesterterpene constituents on this effects, erinacine A and S were isolated and their effects on attenuating AD-related pathology in APPswe/PS1dE9 transgenic mice were investigated. A 30 day short-term administration of erinacine A and S were performed to explore the effect of each erinacine on AD-related pathology including amyloid β production and degradation, plaque formation, plaque growth, glial activation and neurogenesis deterioration. Our results indicated the benefit effects of both erinacine A and S in cerebrum of APPswe/PS1dE9 mice, including: (1) attenuating cerebral plaque loading by inhibiting plaque growth; (2) diminishing the activation of glial cells; (3) raising the level of insulin degrading enzyme; and (4) promoting hippocampal neurogenesis. Moreover, erinacine A reduced the level of insoluble amyloid β and C-terminal fragment of amyloid precursor protein which was not mediated by erinacine S. We further performed a long term administration of erinacine A and found that erinacine A recovered the impairment in the tasks including burrowing, nesting, and Morris water maze. Our data pointed out that although both erinacine A and S reduce AD pathology via reducing amyloid deposition and promoting neurogenesis, erinacine A can also inhibit amyloid β production and is worth to be further developed for AD therapeutic use.

## 1. Introduction

Alzheimer’s disease (AD) is characterized by progressive cognitive decline, neurofibrillary tangles, amyloid plaques, neuro-inflammation, and decline of adult neurogenesis [1,2,3]. Amyloid β (Aβ), a peptide formed from procession of amyloid precursor protein (APP), is thought to be one of the primary initiating factors in AD pathology. The amyloid hypothesis recommends that AD is caused by an imbalance between production and clearance of Aβ [1], resulting in increased amounts of Aβ in monomeric, oligomeric, and insoluble fibrillary forms in the Central Nervous System (CNS) and which subsequently induces Aβ plaque formation, neuroinflammation, and oxidative stress [4].

Increasing evidence has indicated that adult hippocampal neurogenesis play the major role in cognitive function [5]. The APP metabolites, including oligomeric Aβ, soluble APPα (sAPP)α, and APP intracellular domain (AICD), have been found to regulate the properties of human neural stem cells which influence adult hippocampal neurogenesis [6,7]. On the other hand, the proinflammatory cytokines such as IL-1β, TNF-γ, and IL-6 produced by activated glia may also regulate the process of adult hippocampal neurogenesis [8,9,10].

*Hericium erinaceus* is an edible and medicinal mushroom with various pharmacological activities, including anti-neurodegenerative and neuroprotective activities [11,12]. Erinacines isolated from its mycelia have been known to possess a potent stimulating effect on nerve growth factor (NGF) expression and secretion [13]. Recent studies have demonstrated that *H. erinaceus* fruiting body ameliorated Aβ-induced cognitive declined in mice and people with mild cognitive impairment [14,15]. Our previous studies also demonstrated that *H. erinaceus* mycelium ameliorated Aβ-induced cognitive decline in mice [16]. The major components of *H. erinaceus* mycelium are erinacine A (HE-A), C (HE-C), and S (HE-S) which are belong to cyanthin diterpenoid (both HE-A and HE-C) and sesterterpene (HE-S). For verifying the effect of these different constituents of *H. erinaceus* mycelium on AD-related pathologies, HE-A and HE-S were isolated and their effects were compared in the present study.

The APPswe/PS1dE9 mouse model (APP/PS1), co-expressed Swedish, mutated human APP695 and human mutated presenilin 1 (PS1) in which exon 9 is deleted [17], exhibit AD-like pathological and behavioral changes, including accumulation of amyloid plaques in brain, degeneration of cholinergic system, and impaired exploratory behavior and spatial memory [18]. Increased Aβ production and plaque formation in APP/PS1 mice has been shown to occur as early as 3 to 5 months-old [19], and impaired spatial learning and memory was observed at six months-old [20,21]. Furthermore, the neurogenesis is also found to be impaired in the APP/PS1 mouse at 3 to 6 months-old [22].

In the present study, the potentials of HE-A and HE-S on amyloid pathology in 5 months old APP/PS1 mice were investigated and compared. Our data suggests that although both HE-A and HE-S were active on reducing Aβ plaque growth, diminishing neuroinflammation and increasing the level of insulin-degrading enzyme (IDE), only HE-A reduced the level of insoluble amyloid β and APP C-terminal fragment (CTF) and decreased the initiation of Aβ formation.

## 2. Results

### 2.1. Experimental Design

**Figure 1:**
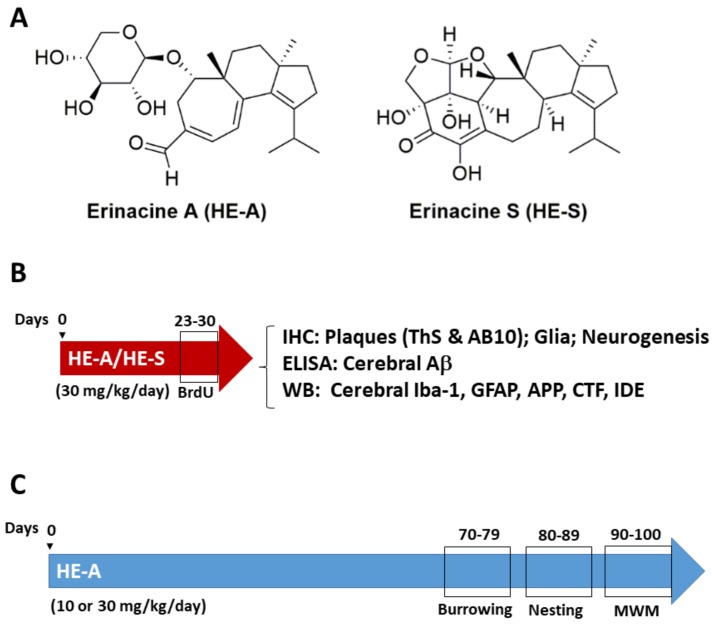
The structure of erinacine A (HE-A) and erinacine S (HE-S) and experimental design. (**A**) The structure of HE-A and HE-S were shown; (**B**) Short-term administration: Five month old female amyloid precursor protein (APP)/human mutated presenilin 1 (PS1) mice were orally administered with vehicle, HE-A and HE-S for 30 days (*n* = 8 for each group). BrdU was injected intraperitoneally at the last 7 days of drug administration for detecting neurogenesis. The mice were scarified and the indicated assays were performed; (**C**) Long-term administration: Five months old APP/PS1 mice were orally administered with vehicle or HE-A for 100 days (*n* = 8 for each group). The tasks of burrowing, nesting, and Morris water maze (MWM) were initiated at 70th, 80th, and 90th day after orally administration, respectively.

APP/PS1 transgenic mice was used as AD animal model to examine the effects of HE-A and HE-S (Figure 1A) on ameliorating AD-related pathologies. For studying the therapeutic effect in short-term administration, female APP/PS1 mice were administrated by gavage with vehicle (*n* = 8), HE-A or HE-S (30 mg·kg^−1^·day^−1^, *n* = 8 for each group) at 5 months of age for 30 days. For control, female Wild type mice (WT) were administrated by gavage with vehicle (*n* = 5) at 5 months of age for 30 days. Male mice were also used to verify the difference between male and female, and no significant difference was observed. BrdU (5-bromo-2′-deoxyuridine) was injected intraperitoneally at 50 mg·kg^−1^·day^−1^ during the last 7 days. The detail Aβ-related pathological changes in mice brain were examined using immunohistochemistry, enzyme-linked immunosorbent assay (ELISA) and immunoblotting (Figure 1B). For behavior effect assays, the experiment is designed to the long-term administration of vehicle (*n* = 8) or HE-A (10 or 30 mg·kg^−1^·day^−1^, *n* = 8) to APP/PS1 mice at 5 months of age for 100 days (Figure 1C). For control, female Wild type mice (WT) were administrated by gavage with vehicle (*n* = 5) at 5 months of age for 100 days.

Burrowing task was performed during the time between the 70th and 79th day after drug administration; nesting task was performed during the time between the 80th and 89th day after drug administration; Morris water maze was performed during the time between the 90th and 100th day after drug administration. The animal were sacrificed at 100th day after drug administration and then the Aβ-related pathological changes were inspected by immunoblotting.

### 2.2. Both Erinacine A (HE-A) and Erinacine S (HE-S) Reduced Aβ Plaque Burden and Elevated Aβ Degradation in Cerebrum of Amyloid Precursor Protein (APP)/Human Mutated Presenilin 1 (PS1) Mice

**Figure 2:**
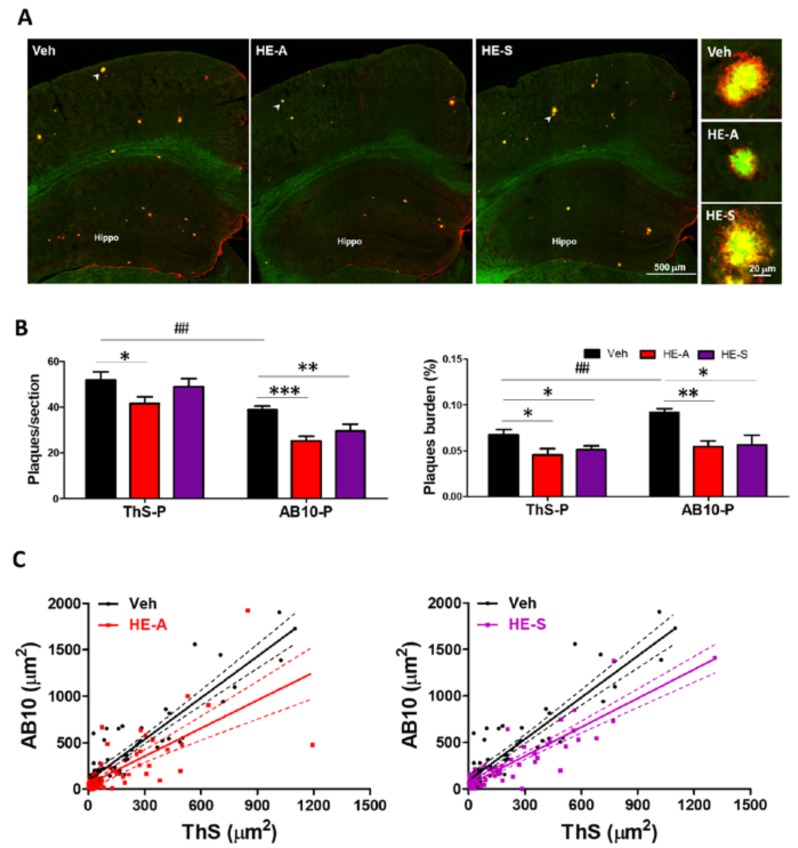
HE-A and HE-S reduce amyloid plaque burden and size in APP/PS1 mice. APP/PS1 transgenic mice orally administered with vehicle (Veh) and HE-A or HE-S for 30 days (*n* = 8 for each group), and then amyloid plaques were stained by thioflavin S (ThS) and immuno-stained with AB10 antibody. (**A**) The representative fluorescent images of ThS (green) and AB10 (red) in the indicated area were shown. Hippo, hippocampus. Scale bar: 500 μm. The typical plaques (arrow) are magnified and is shown at the right side. Sale bar: 20 μm; (**B**) The number and burden of ThS- stained plaque (ThS-P) and AB10-stained plaque (AB10-P) in cerebral hemisphere were calculated by image analysis software. Plaque burden is displayed as a percentage of the area occupied by ThS or AB-10 stained signal in the full area of interest. The results are the mean ± standard error of mean (S.E.M). Significant differences between Veh group and the other groups are indicated by *, *p* < 0.05; **, *p* < 0.01; ***, *p* < 0.001. Significant differences between 30 d-ThS group and the other groups are indicated by ##, *p* < 0.01; (**C**) Scatter plots of ThS- and AB10-stained area of each single plaque in the brain slice. (Solid lines: linear regression lines; dashed lines: 95% confidence intervals).

It is well established that plaques are visible in 6 month old APP/PS1 mice [23]. Therefore, 5 month old APP/PS1 mice orally administrated with vehicle or 30 mg·kg^−1^·day^−1^ of HE-A and HE-S for 30 days were employed to explore the effects of HE-A and HE-S on Aβ plaque deposition (Figure 2). The body weight changes was examined during erinacine administration, and no significant different in body weight were observed among experimental groups.

To understand the three-dimentional structure of amyloid plaque in brain of APP/PS1 mice, both ThS and AB10 antibody were used to stain the amyloid plaque. In a previous study, ThS stained the compact core region with β-structure (ThS-P) and AB10 imunostained the peripheral region without β-sheet structure (AB10-P) [16]. The plaque burden was calculated as the percentage of total plaque area in the observed brain area to detect the change in both number and size of plaque. ThS-P and AB10-P occupied 0.07 ± 0.01% and 0.09 ± 0.01% of the observed brain area, respectively, in vehicle group (Figure 2B). The administration of HE-A and HE-S significantly reduced the burden of ThS-P. However, the number of ThS-P was only significantly changed by the treatment of HE-A, indicating that the effect of HE-A was on both the size and number of ThS-P. However, HE-S only affected the size, but not the number, of ThS-P. On the other hand, both burden and number of AB10-P were significantly reduced after the treatment of both HE-A and HE-S, indicating that both HE-A and HE-S treatments inhibited growth of the peripheral region of AB10-P. To verify this hypothesis, the size alteration of individual plaque co-stained with ThS and AB10 was analyzed by a scatter plot (Figure 2C). Linear regression analysis revealed that the slope of regression equation of both HE-A and HE-S groups were significantly different from the vehicle group. Linear regression for vehicle group is *y* = 1.49*x* + 90.55, *R*^2^ = 0.82; for HE-A group is *y* = 1.01*x* + 54.23, *R*^2^ = 0.51 (*p* < 0.01 for difference in slopes); and for HE-S group is *y* = 1.03*x* + 44.84, *R*^2^ = 0.80 (*p* < 0.001 for difference in slopes). This result indicates that both HE-A and HE-S eliminated the peripheral region of AB10-P, but not the compact core region (i.e., central region of AB-10-P and whole ThS-P). We have also performed immunohistochemical analysis on long term animals, and similar results were observed.

### 2.3. HE-A, but Not HE-S, Decreased Aβ Accumulation by Inhibiting Aβ Production in Cerebrum of APP/PS1 Mice

**Figure 3:**
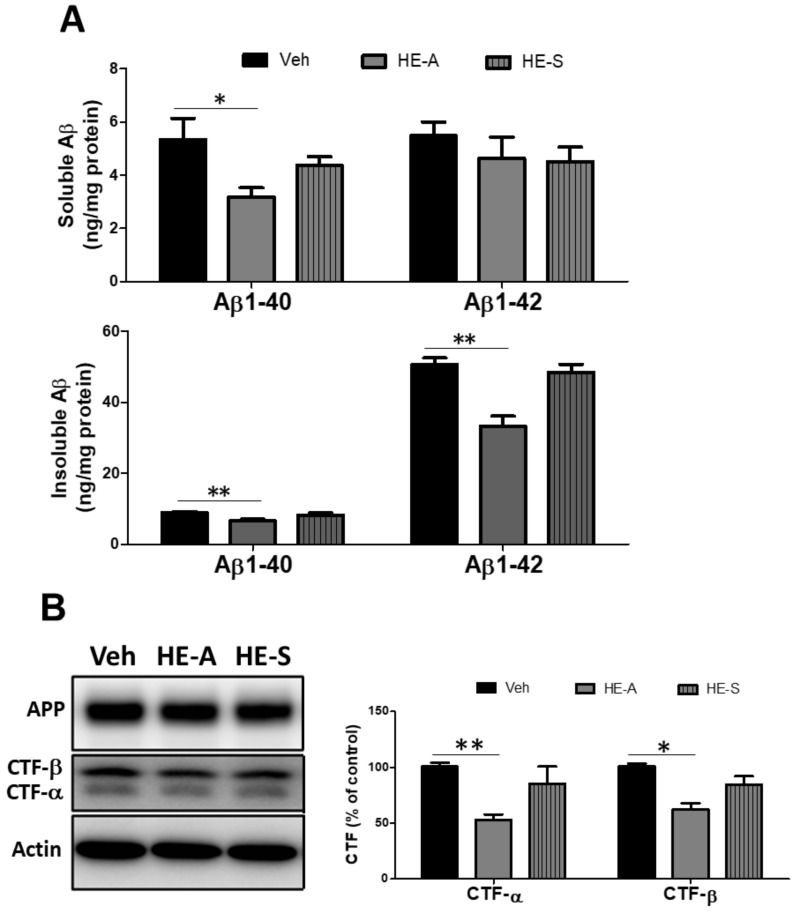

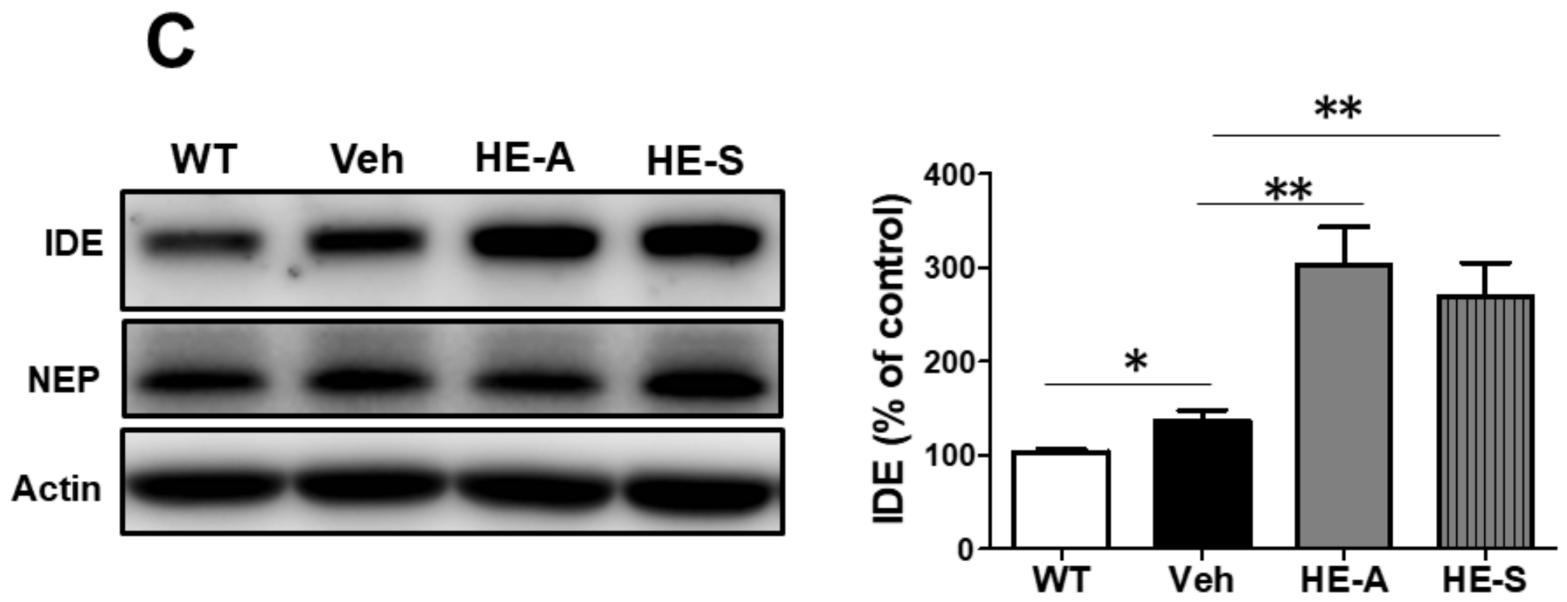
HE-A, but not HE-S, reduces Aβ accumulation in APP/PS1 mice. APP/PS1 transgenic mice were orally administered with vehicle (Veh), HE-A or HE-S for 30 days (*n* = 8 for each group), and then the level of Aβ_1−40_ and Aβ_1−42_ in cortical homogenates was determined by enzyme-linked immunosorbent assay (ELISA) (**A**). The level of APP and C-terminal fragment (CTF) (**B**), and insulin-degrading enzyme (IDE) and neprilysin (NEP) (**C**) in homogenates were analyzed by immunoblotting. The level of IDE and NEP in wild type (WT) mice (*n* = 5) is also compared. Representative immunoblots are shown at the left panel. The ratio of CTF-α and −β to β-actin is presented as percentage of Veh group. The ratio of IDE to β-actin is presented as percentage of WT group. The results are the mean ± S.E.M. Significant differences between Veh group and the other groups are indicated by *, *p* < 0.05; **, *p* < 0.01.

The above result indicated that the number of ThS-P was eliminated by HE-A, but not HE-S (Figure 2B). Therefore, we hypothesize that HE-A, but not HE-S, decreased Aβ accumulation by inhibiting Aβ production. To confirm this hypothesis, the level of Aβ in the cerebral cortex was determined using ELISA analysis (Figure 3A). The result showed that administration of HE-A significantly reduced the level of SDS-insoluble Aβ_1–40_ and Aβ_1–42_ and SDS-soluble Aβ_1–40_ in the cerebral cortex. Nevertheless, the administration of HE-S had no significant effect on the level of both SDS-soluble and SDS-insoluble Aβ. To determine the effect of HE-A and HE-S on Aβ production, the levels of CTF-β, the precursor of Aβ, were detected by Western blotting. Again, the result showed that administration of HE-A, but not HE-S, significantly reduced the levels of CTF-β (Figure 3B). Moreover, the level of CTF-γ was also reduced by HE-A suggesting that HE-A may inhibit the activity of both α- and β-secretase.

The above results indicated that both HE-A and HE-S reduced Aβ plaque burden. Thus, we hypothesize that both HE-A and HE-S may promote Aβ clearance by protein degradation. To verify this hypothesis, the level of Aβ degrading enzymes, such as IDE and neprilysin (NEP), were detected by immunoblot. We found that the level of cortex IDE in the vehicle-treated trangenic mice (Veh group) is significantly increased to 136.6% of WT group. The level was further increased to 303.5% and 269.8% of WT group after treated with HE-A and HE-S, respectively (Figure 3C). On the other hand, no significant change of NEP was observed after treated with HE-A and HE-S. We also performed IDE level analysis on long term animals, and similar results were observed

### 2.4. Both HE-A and HE-S Reduce Glial Activation in Cerebrum of APP/PS1 Mice

Next, we evaluated the effects of HE-A and HE-S on activation of microglia and astrocytes. To determine the both plaque-dependent and independent activations of microglia and astrocytes in the cerebrum of APP/PS1 transgenic mice, Aβ plaque, microglia and astrocytes were examined by immunostaining of AB10, Iba-1, and glial fibrillary acidic protein (GFAP), respectively. We found that plaque-associated microglia in the cerebral cortex immersed their processes into AB10-P and contacted with the ThS-P. Likewise, the plaque-associated astrocytes extended their processes to contact AB10-P. By contrast, the non-plaque-associated microglia showed more ramified morphology indicating their resting state, and the non-plaque-associated astrocytes hardly express GFAP.

**Figure 4:**
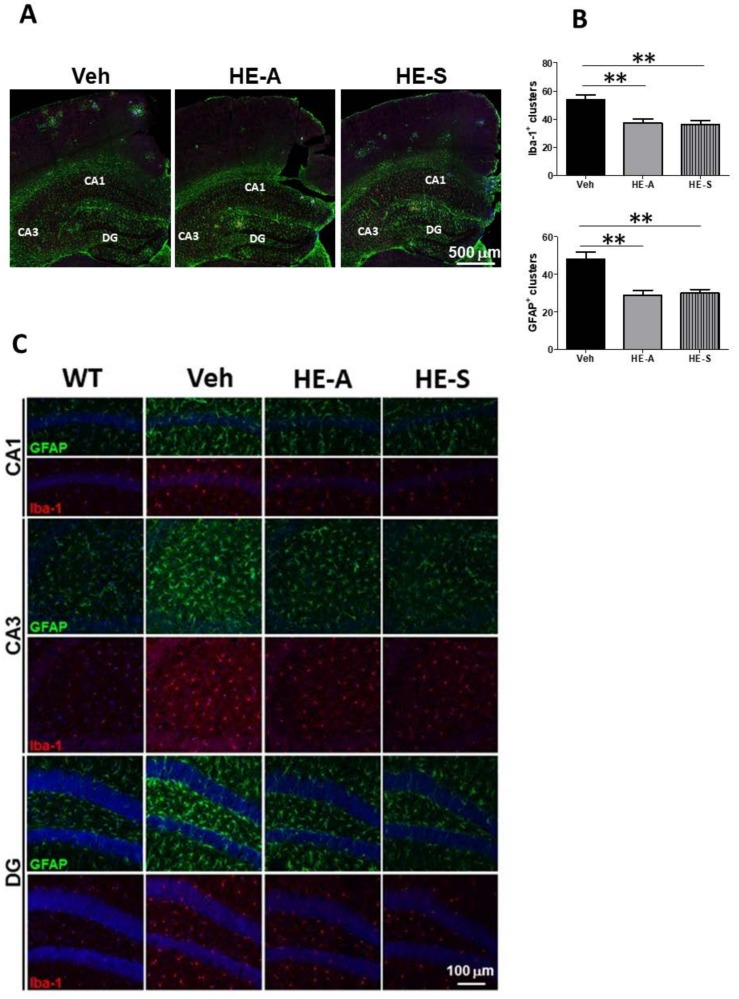

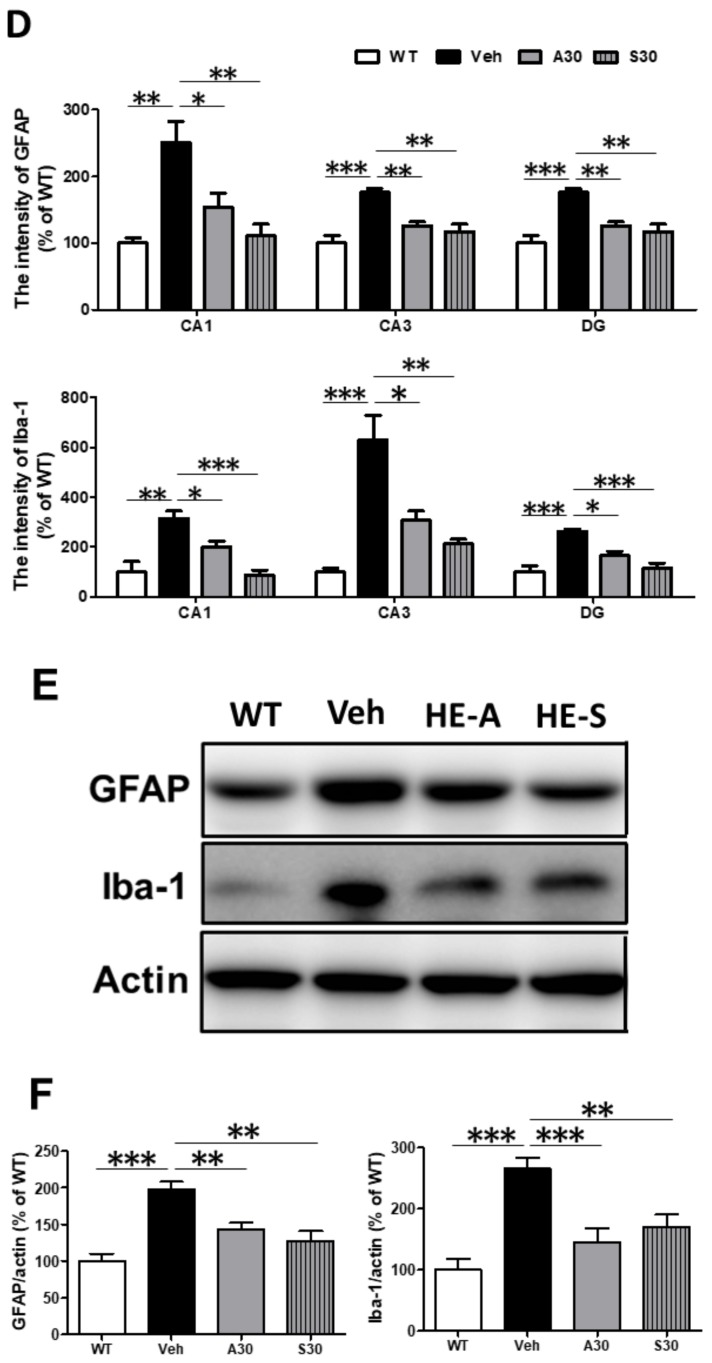
HE-A and HE-S reduce glial activation in APP/PS1 mice. APP/PS1 transgenic mice were orally administered with vehicle (Veh), HE-A or HE-S for 30 days (*n* = 8 each). Amyloid plaques were detected by immunohistochemical staining with AB-10 antibody (blue). Microglia and reactive astrocytes were detected by immunohistochemical staining with Iba-1 antibody (red) and glial fibrillary acidic protein (GFAP) antibody (green), respectively. The representative immunostaining images of clusters in section of parietal cortical area and temporal cortical area are shown (**A**). Scale bar: 500 µm. The number of microglial and astroglial cluster in the section of cerebral hemisphere is calculated by image analysis software and shown in (**B**). The representative immunostaining images of glial cells without associated with plaque in Cornu Amonis (CA)1, CA3, dentate gyrus (DG) were shown in (**C**). The image in WT mice (*n* = 5) is also compared. Sale bar: 100 µm. The immunoreactivity of Iba-1 and GFAP in the CA1, CA3, and DG is calculated by image analysis software and shown in (**D**). The level of GFAP and Iba-1 in homogenates were analyzed by immunoblotting (E, F). The protein level in WT mice (*n* = 5) is also compared. Representative immunoblots are shown at (E). The ratio of CTF-α and −β to β-actin is presented as percentage of Veh group. The ratio of IDE to β-actin is presented as percentage of WT group. The results are the mean ± S.E.M. Significant differences between Veh group and the other groups are indicated by *, *p* < 0.05; **, *p* < 0.01; ***, p < 0.001.

There is about 50 plaque-associated glial clusters in a section of hemi-cerebral sphere been observed, and both HE-A and HE-S treatments decreased the number of plaque-associated microglial and astroglial clusters (Figure 4A,B). Moreover, the immunoreactivity of the non-plaque-associated glial cells in hippocampus of various groups were also compared (Figure 4C,D). The results showed that immunoreactivity of both Iba-1 and GFAP are enhanced in vehicle-treated APP/PS1 mice as compared with WT mice, which are significantly ameliorated by the treatment of both HE-A and HE-S (Figure 4C,D). The activation of microglia and astrocytes were further confirmed by immunoblotting of Iba-1 and GFAP. The results showed that the level of both Iba-1 and GFAP are higher in vehicle-treated APP/PS1 mice as compared with WT mice, and which are decreased by the treatment of both HE-A and HE-S. (Figure 4E,F).

### 2.5 Both HE-A and HE-S Promote Hippocampal Neurogenesis and Dendritic Complexity in APP/PS1 Mice

**Figure 5:**
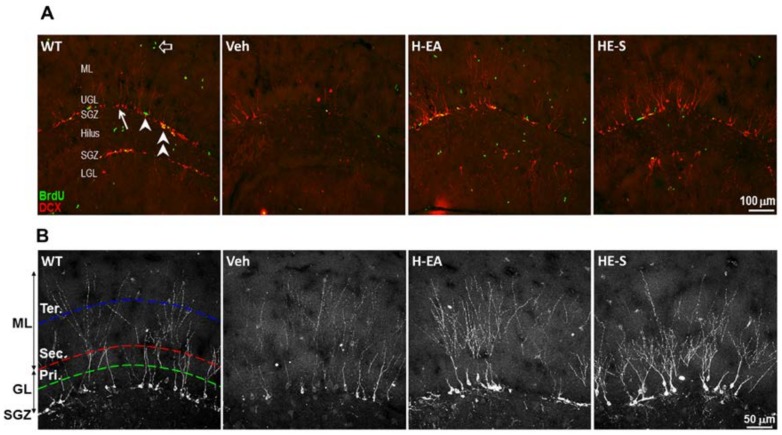

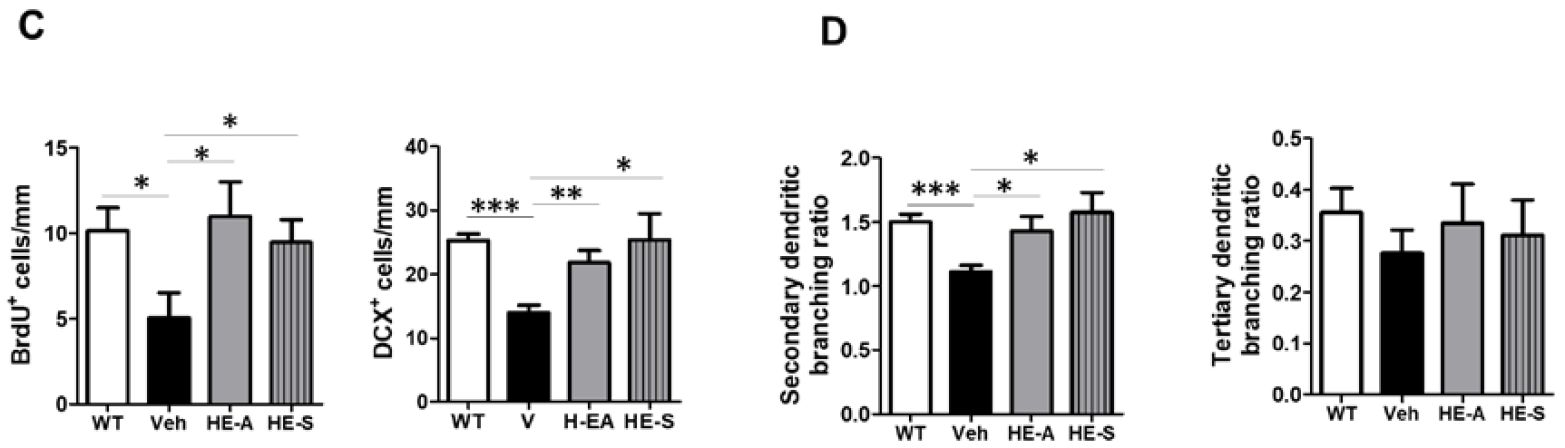
HE-A and HE-S promote hippocampal neurogenesis in APP/PS1 mice. APP/PS1 transgenic mice were orally administered with vehicle (Veh), HE-A or HE-S for 30 days (*n* = 8 each). WT mice were used as non-transgenic control. Hippocampal neurogenesis was detected by immunohistochemical staining with doublecortin antibody (DCX, red) and BrdU antibody (green). The representative immunostaining images of the dentate gyrus area are shown in (**A**). Arrow indicates DCX-labeled newly born neuron; arrow head indicates proliferating type 2 neuroprogenitor; double arrow head indicates the newly born neuron immediately after proliferation; hollow arrow indicates proliferating cells other than neuroprogenitor. ML, molecular layer; UGL, upper blade granular cell layer; SGZ, subgranular zone; LGL, lower blade granular cell layer. Scale bar: 100 µm. The representative immunostaining images of the upper blade dentate gyrus area are shown in (**B**). Scale bar: 50 μm. Primary (pri.), secondary (sec.) and tertiary (ter.) dendrites of the DCX^+^ cells are counted along the middle of granular cell layer (GCL) (green dashed line), the outer edge of GCL (red dashed line), and the middle of ML (blue dashed line), respectively. (**C**) The number/mm SGZ of BrdU positive cells (BrdU^+^); doublecortin positive cells (DCX^+^) and the cells with double labeling (BrdU^+^, DCX^+^). (**D**) The dendritic complexity was analyzed by laminar quantification. The branching ratio of secondary and tertiary dendrites of the DCX^+^ cells were shown. Dendrites of the DCX^+^ cells are counted along the middle of GCL, the outer edge of GCL, and the middle of ML, respectively. The results are the mean ± S.E.M. Significant differences between Veh group and the other groups are indicated by *, *p* < 0.05; **, *p* < 0.01; ***, *p* < 0.001. Results were analyzed by Student’s *t* test.

We hypothesized that the erinacine-mediated decline of AB-10-P burden and microglial activation may subsequently promote hippocampal neurogenesis. To certify this hypothesis, we assessed newly born granular neurons and proliferating type 2 progenitors in the subgranular zone (SGZ) by immunohistochemical staining using anti-doublecortin (DCX) and anti-BrdU antibody, respectively. There is a decline of the number of DCX-positive newly born granular neurons and BrdU-positive proliferating type 2 progenitors in SGZ of APP/PS1 mice as compared with that of WT mice, and which were recovered by treatment of HE-A and HE-S (Figure 5A,C).

Because the dendrite growth is important for neuronal integration in neurogenesis, we further analyzed the dendritic complexity of DCX positive cells by laminar quantification method [24]. We found that the level of secondary dendritic branching, but not the level of primary and tertiary branching, is less in vehicle treated transgenic mice as compared with WT mice. HE-A and HE-S significantly increased the dendritic complexity in secondary dendritic branching (Figure 5B,D).

### 2.6. HE-A Recovers the Cognitive Decline in APP/PS1 Mice

**Figure 6:**
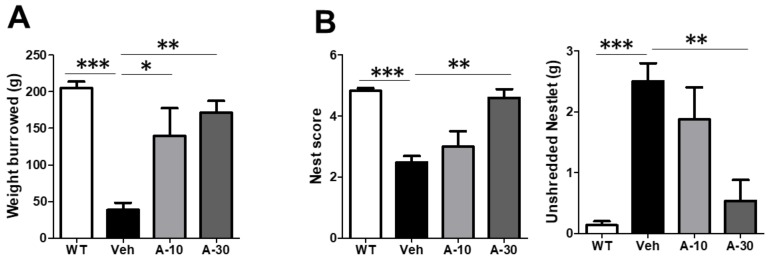

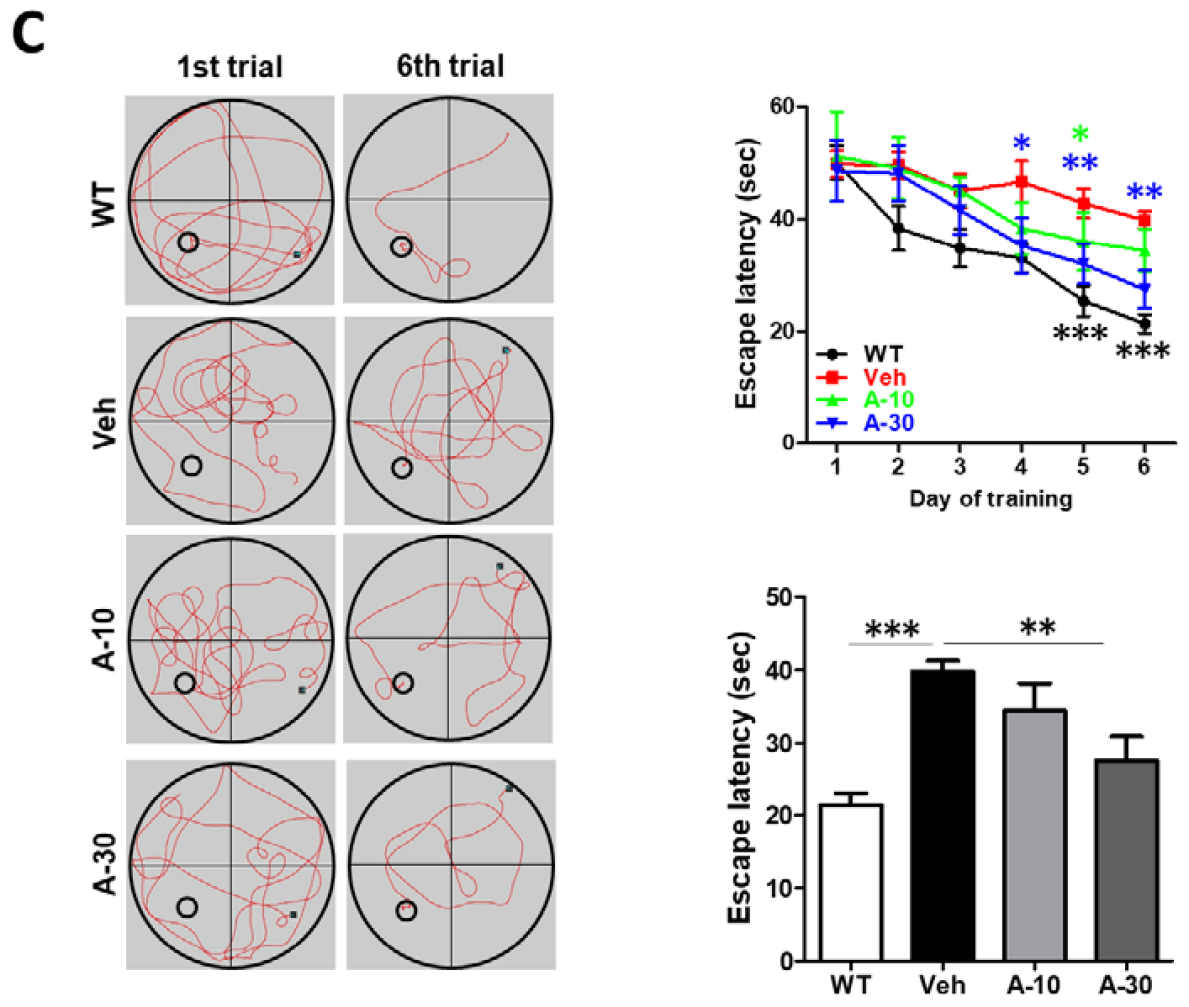
HE-A ameliorates behavior deficits in APP/PS1 mice. APP/PS1 transgenic mice were orally administered with vehicle (Veh) or HE-A at 10 µm (A-10) and 30 µm (A-30) (*n* = 8 each). The tasks of burrowing and nesting were performed at 70 and 80 days, respectively, post administration. Wild type mice treated with vehicle (WT) was used as non-transgenic control. APP/PS1 mice treated with vehicle (Veh) was used as non-medication control. Bar graphs show the results from burrowing task (**A**), and the nesting task’s nest score and unshredded Nestlet (**B**) from nesting task. MWM were performed. Wild type mice treated with vehicle (WT, *n* = 5) was used as non-transgenic control. (**C**) Morris water maze (MWM) was performed. The representative swim paths in the hidden platform tests at 1st and last trial (**left** panel). Escape latency during the training phase (**up right** panel) and at the 6th day of training (**low right** panel) was examined. The results are the mean ± S.E.M. Significant differences between Veh group and the other groups are indicated by *, *p* < 0.01; **, *p* < 0.01; ***, *p* < 0.001. Groups labeled with different letters in escape latency were significantly different from each other with two way ANOVA, *p* < 0.05.

Burrowing and nesting behaviors, which engage a broad network of brain regions, have previously been applied on evaluating the activities of daily living (ADL) skills of AD transgenic mice. In the present study, five month old APP/PS1 mice were orally administered with HE-A (10 and 30 mg·kg^−1^·day) for 100 days. The tasks of burrowing and nesting were then initiated at the 70th and 80th day after orally administration, respectively (Figure 6A). APP/PS1 mice showed deficit in spontaneous burrowing behavior which was significantly recovered by administrating HE-A at both 10 and 30 mg·kg^−1^. APP/PS1 mice also showed deficit on nesting behavior evaluated by nest score and unshredded nestlet. The impaired nesting behavior was significantly recovered by administration of HE-A at 30 mg·kg^−1^ dose, but not at 10 mg·kg^−1^ dose (Figure 6B). In MWM task, APP/PS1 mice showed a longer escape latency to find the hidden platform than WT mice during the training phase, suggesting that APP/PS1 mice showed a spatial learning impairment at 8 months-old. This obvious impairment was significantly recovered by the treatment of HE-A at 30 mg·kg^−1^ dose, but not at 10 mg·kg−^1^ dose (Figure 6C). Two way repeated measurement analysis of variant (ANOVA) analysis confirmed an interaction between groups and days of training on Escape latency to find the plateform (*F* = 3.224, *p* = 0.0001). It is significantly different among the days of training (*F* = 20.35, *p* < 0.0001), among groups (*F* = 10.21, *p* = 0.0001), and in subject (*F* = 1.574, *p* = 0.0458). In Bonferroni posttests, it is significantly different between WT and vehicle groups at the fifth (*t* = 5.570, *p* < 0.001) and sixth (*t* = 5.394, *p* < 0.001) day of training. It is significantly different between vehicle and A-10 groups at the fifth day of training (*t* = 2.810, *p* < 0.05). It is significantly different between vehicle and A-30 groups at the fourth (*t* = 2.789, *p* < 0.05), fifth (*t* = 3.619, *p* < 0.01) and sixth (*t* = 3.408, *p* < 0.01) day of training.

**Figure 7:**
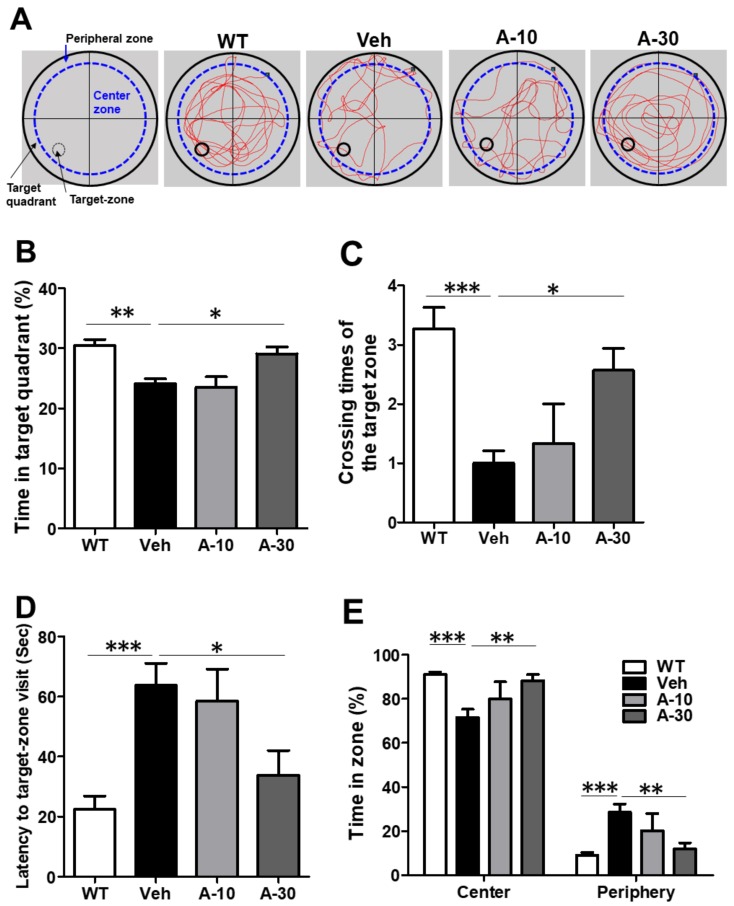
HE-A improved the spatial learning and memory during probe trial in APP/PS1 mice. APP/PS1 transgenic mice were orally administered with vehicle (Veh) or HE-A at 10 µm (A-10) and 30 µm (A-30) (*n* = 8 each). The representative swim path in a probe trial tests (**A**). A-30 group significantly reverse the memory retention deficit by prolonging the time in target quadrant (**B**), crossing times of the former platform (**C**), latency to target-zone visit (**D**), and the percentage of time spent in the center vs. periphery zones in the probe trial (**E**). The results are the mean ± S.E.M. Significant differences between Veh group and the other groups are indicated by *, *p* < 0.05; **, *p* < 0.01; ***, *p* < 0.001.

In a probe trial, APP/PS1 mice showed reductions in the percent time spent in the target quadrant, crossing time in target zone and latency to target zone visit (Figure 7A–D) without affecting the swing speed. Again, this impairment was significantly recovered by the treatment of erinacine A at 30 mg·kg^−1^ dose, but not at 10 mg·kg^−1^ dose. It is worth noting that APP/PS1 mice showed a stereotyped behavior spending more time in the periphery of the water maze pool than WT mice during probe trial (Figure 7A,E). Whereas HE-A treatment reversed this stereotyped behavior in the periphery to the control level at 30 mg·kg^−1^ dose, but not at 10 mg·kg^−1^ dose.

## 3. Discussion

**Figure 8:**
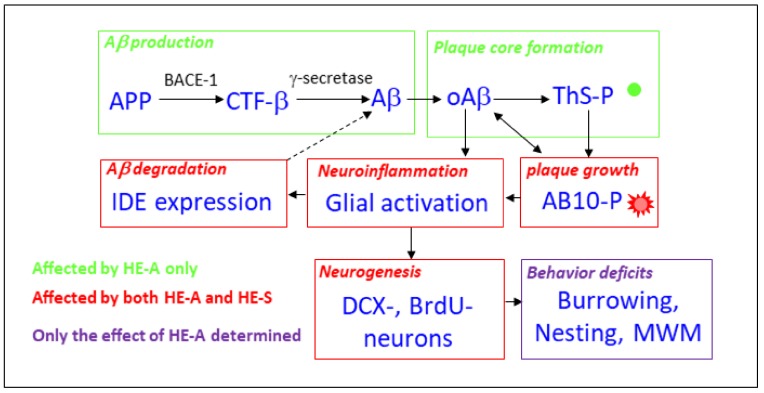
Schematic diagram of the pathogenic pathway of AD in APP/PS1 transgenic mice and the effects of HE-A and HE-S. This schematic diagram summarize the potential pathogenic pathway of AD in APP/PS1 transgenic mice. Altered APP processing cause Aβ accumulation, oligomerization (oAβ) and plaque core formation (ThS-P). Plaque can grow overtime with the large, diffuse Aβ positive plaque ring-like structure (AB10-P). There is a putative balance between oAβ and AB10-P, both of which may leading to neuroinflammation. Neuroinflammation reduce hippocampal neurogenesis and lead to the behavior deficits. The pathway affected by HE-A only and both HE-A and HE-S are indicated in green and red color, respectively. On behavior tests, only the effect of HE-A was examined (in purple).

The effects of HE-A and HE-S on the sequence of major pathogenic events of AD in APP/PS1 transgenic mice is depicted in Figure 8. Altered APP processing by APP/PS1 mutation cause the accumulation of monomeric and oligomeric Aβ, which form plaque core as detected by ThS-staining (ThS-P). ThS-P can grow overtime with the large, diffuse Aβ positive plaque ring-like structure as detected by AB-10-immunostaining (AB10-P). Putatively, both oAβ and AB10-P caused neuroinflammation and the subsequent impairment of hippocampal neurogenesis and then lead to the behavior deficits. Both HE-A and HE-S were effective on suppressing plaque growth, reducing neuroinflammation, and promoting Aβ degradation by enhancing IDE expression, and increasing neurogenesis. Alternatively, HE-A, but not HE-S, reduced Aβ production and plaque core formation, which suggest that HE-A is better than HE-S to be developed for AD therapy. Hence, the effect of HE-A on ameliorating behavior impairment was examined, and the result found that HE-A recovered all three tasks detected in APP/PS1 mice.

The dosages selected was based on the previous study, which has indicated that the no-observed-adverse-effect-level of HE-A-enriched *H. erinaceus* is greater than 3 g·kg^−1^·da−^1^ in Sprague-Dawley rats [25]. The HE-A content of the same HE-A-enriched *H. erinaceus* preparation is 19 mg·g^−1^. Therefore, the biosafety of purified HE-A could be greater than 57 mg·kg^−1^·day^−1^ in APP/PS1 mice. Hence, 10 and 30 mg·kg^−1^·day^−1^ were selected as our experimental dosages.

HE-A, but not HE-S, reduced the level of SDS-soluble Aβ1-40, SDS-insoluble Aβ, and CTF-β in cerebral cortex suggesting that Aβ production was inhibited by HE-A, but not by HE-S. As comparing the isoforms of the SDS-insoluble Aβ, we found that HE-A selectively decreased the level of the most aggregation-prone Aβ1-42 as compare to Aβ1-40. These results suggest that Aβ aggregation were diminished by HE-A, which may subsequently decrease the initiation of amyloid plaque formation. Recent study has shown the similar effects of anthocyanin-enriched bilberry extract which was found to reduce the level of Aβ by modulating APP processing in APP/PS1 mice [26].

The structure of amyloid plaque has been characterized to be initiated by the dense-core which is surrounded by a diffuse structure formed during plaque growth [27]. These plaque structure was confirmed in our previous study using double staining of ThS and AB-10 antibody [16]. In our present study, we found that HE-A, but not HE-S, caused a decreasing number of ThS-P, but of AB10-P. This result suggested that the initiation step of plaque formation was inhibited by HE-A, but not by HE-S. On the other hand, both HE-A and HE-S significantly decreased the plaque burden, but not the plaque number, of AB-10-P in APP/PS1 mice, suggesting that growing step of plaque formation was affected by both HE-A and HE-S. We further compared the sized of each single plaque which has been simultaneously stained by AB10 and ThS in a scatter plot to verify the effect erinacines on the growing step of plaque formation, and found that the size ratio of each plaque stained by AB-10 and THS-P was significantly reduced after HE-A and HE-S treatment. Previous studies found that endogenous antibody delivery reduced plaque size by activating microglia to internalize Aβ [28]. Therefore, the same mechanism of erinacine to reduce plaque sized may be hypothesized.

Recent evidence suggests that impaired clearance may be the driving force behind sporadic AD [29]. Microglia may contribute to Aβ clearance through phagocytosis or the activity of proteolytic enzymes, including IDE and NEP [30,31]. IDE knockout mice and rat with partial loss-of-function mutations in IDE increased cerebral Aβ accumulation by 50% [32]. In contrast, previous work demonstrated that two-fold increase of IDE via transgenic over-expression reduces obviously 50% Aβ deposition in APP transgenic mice [33]. Moreover, both Aβ and AICD are the substrates of IDE [34] which is located in the cytosol and a small proportion of IDE also secreted out from cells [30]. Therefore, it is likely that the increase of IDE expression may attenuate amyloid plaque burden. In the present study, we found that both HE-A and HE-S promoted IDE expression, suggesting that Aβ clearance by degradation may be involved in the Aβ reduction effect.

The expression levels of IDE were age-dependently up-regulated in APPswe/PSEN1(dE9) mice, APPswe/PSEN1(A246E) mice, and Tg2576 mice in parallel with Aβ accumulation [35,36]. Therefore, additional IDE may derive from the result of Aβ-triggered neuroinflammation [36]. In APP/PS1 mice, the increased expression of IDE was also observed in our present study at 6 months-old. By contrary, another study found that the brain level of IDE decline significantly in ApoE4 allele associated AD [37]. In APP/PS1 mice, the declined expression of IDE was also observed in our unpublished study at 8 months-old. Therefore, the decline of brain IDE at later stage of AD progression may be the important factor on increasing the level of cerebral Aβ in AD, and which will be a therapeutic choice for AD. Although PPAR-γ and Notch signaling have been shown to enhance IDE expression [36,38], the mechanism for HE-A to promote IDE expression remains to be explored.

While it is clear that astrocytes and microglia cluster around amyloid plaques in AD, whether they are primarily attracted to amyloid deposits or are just reacted to plaque-associated damages neurites remains elusive [39]. Microglial activation is highly associated with the accumulation of Aβ since that activated microglia are found to surround plaque. Thereby, activated microglia may clear Aβ through phagocytosis or proteolytic degradation [40]. The functional impact of phagocytosis is highlighted by studies showing that restricting microglial accumulation and phagocytosis increases Aβ deposition [41].

In the present study, we found that HE-A and HE-S promote neurogenesis. APP could influence neurogenesis via sAPPβ and AICD [42]. Administration of HE-A, but not HE-S, significantly decreased the level of both CTF-α and CTF-β, which are precursors of AICD. Therefore, neurogenesis may be promoted by the decrease of AICD after the administration of HE-A. Furthermore, inflammatory challenge triggered by Aβ induces the production of proinflammatory cytokines by microglia as well as astrocytes have profound detrimental effects on adult neurogenesis (8).

A previous study found that APP/PS1 transgenic mice increased Aβ production and plaque formation at around 3–5 months of age [43]; developed mild impairments of spatial memory and nesting skill at 4.5 months of age [44]; and displayed cognitive deficits in the MWM [45]. In the present study, 5 month old APP/PS1 mice were employed and oral administration of HE-A at 10 or 30 mg·kg^−1^·day^−1^ for 100 days and the behavior assays of burrowing, nesting and MWM were performed started at 7.3, 7.6, and 8.0 months old, respectively.

To verify the effects of HE-A on behavior impairment in APP/PS1 mice, we focus on species-specific burrowing and the nest building activity because those are spontaneous multiple brain regions-dependent behaviors [46,47], and which have been proposed to be equivalent to activities of daily living (ADL) skills in humans [48]. Clinically, the loss of ADL skills is one of the pathological changes in AD [49], and have been shown in APP/PS1 mice [44,50]. Besides, we also assessed hippocampal-dependent spatial memory formation in APP/PS1 mice using MWM test. Moreover, a stereotyped behavior tending to navigate in the periphery of the pool has been shown in several AD transgenic mice including APP/PS1 mice [51]. It is speculated that this stereotyped behavior reflecting the increased anxiety of APP/PS1 mice, and which might be used as an important behavioral indicator in analysis of transgenic mouse models of AD. In the present work, we found that HE-A attenuate the deficit in burrowing behavior and nesting behavior, spatial learning and memory, and anxiety-related stereotyped behavior. These results suggest that HE-A administration may be potential to restore the impairment in multiple brain regions of APP/PS1 mice.

## 4. Materials and Methods

### 4.1. Materials

BrdU, formic acid, and Thioflavin S were purchased from Sigma-Aldrich (St. Louis, MO, USA). General chemicals were purchased from Sigma-Aldrich (St. Louis, MO, USA) or Merck (Darmstadt, Germany).

### 4.2. Extraction and Isolation of Erinacines

Erinacines were prepared from the ethanol extract of *H. erinaceus* mycelia. Mycelia (2 kg) were refluxed with 95% ethanol. The ethanol solution was concentrated in vacuum to give a brown extract which was partitioned with H_2_O/ethyl acetate (EtOAc) (1:1) to afford H_2_O layer and EtOAc layer. The EtOAc layer was chromatographed on a silica gel column (70~230 mesh, 70 × 10 cm), and eluted with a gradient system of *n*-hexane/EtOAc (10:1; 3:1; 3:2; 1:1; 1:2; 0:1) to give seven fractions (Fr. I−VII). Fraction III, the elute of n-hexane-EtOAc (3:2), was rechromatographed on Sephadex LH-20 and silica gel columns to afford HE-S (525 mg). Fraction VI, the eluate of *n*-hexane/EtOAc (1:2), was separated on a Sephadex LH-20 column eluting with MeOH to yield two sub-fractions (Fr. VI-1 and VI-2). Sub-fraction VI-2 was rechromatographed on Sephadex LH-20 (70% MeOH) and RP-18 (60% MeOH) columns to afford HE-A (8.4 g). The efficiency of extraction of HE-S and HE-A are 525 mg and 8.4 g from 2 kg *H. erinaceus* mycelia, respectively. The identification of HE-A and HE-S in mycelia using NMR and LC-MS-MS have been previously described [16,23,52]. The structure of HE-A and HE-S are shown in Figure 1a.

### 4.3. Management and Administration

The Institutional Animal Care and Use Committee at the National Research Institution of Chinese Medicine approved the animal protocol (IACUC No: 103-417-1 (02.01.2014), 104-417-1 (02.01.2015) and 105-417-1 (02.01.2016)). All experimental procedures involving animal and their care were carried out in accordance with Guide for the Care and Use of Laboratory Animals published by the United States National Institutes of Health (NIH). APP/PS1 was purchased from Jackson Laboratory (No. 005864). Breeding gender ratio was a male with two females in one cage. Experiments were conducted using wild type siblings and AD transgenic female C57BL/6J mice. The animals were housed under controlled room temperature (24 ± 1 °C) and humidity (55–65%) with 12:12 h (07:00–19:00) light-dark cycle. All animal experimental procedures were performed based on Guide for the Care and Use of Laboratory Animals (NIH). APP/PS1 was purchased from Jackson Laboratory (No. 005864). Breeding were conducted using female transgenic mice and the male wild type siblings. The animals were housed under temperature (24 ± 1 °C) and humidity (55%–65%). Light-dark cycle was 12:12 h (07:00–19:00). All mice were provided with commercially available rodent normal chow diet and water ad libitum. For studying the therapeutic effect in short-term administration, female APP/PS1 mice were administrated by oral gavage with vehicle (*n* = 8), HE-A or HE-S (30 mg·kg^−1^·day^−1^, *n* = 8 for each groups) at 5 months of age for 30 days. For behavior effect assays, the experiment is designed to the long-term administration by oral gavage of vehicle, HE-A (10 or 30 mg·kg^−1^·day^−1^, *n* = 8 for each group) to APP/PS1 mice at 5 months of age for 100 days. For control, female Wild type mice (WT) were administrated by gavage with vehicle at 5 months of age for 30 and 100 days (*n* = 5 for each group). BrdU was injected intraperitoneally at 50 mg·kg^−1^·day^−1^ during the last 7 days.

### 4.4. Tissue Processing

At 24 h after the last injection of BrdU, mice were deeply anesthetized with chloral hydrate and then sacrificed by transcardial perfusion with saline (pH 7.4). Mice after anesthetized were sacrificed by transcardial saline perfusion. Mice brain was removed and half brain was homogenized in homogenization buffer containing 20 mM Tris-HCl (pH 7.4), 320 mM sucrose, 2 mM EDTA, 1 mM PMSF, 5 μg·mL^−1^ leupeptin, and 5 μg·mL^−1^ aprotinin. Another half brain was immersed in 4% formaldehyde overnight at 4 °C, and cryoprotected. Then brain tissue was sectioned into 30 μm thick. Three slide spanned approximately bregma −1.58 to −1.82 in each brain were used for staining and analysis.

### 4.5. Thioflavin S Staining

Thioflavin S (ThS; Sigma) is used to detect the deposition of amyloid plaque as described previously [16]. Briefly, dried sections were stained with fresh, filtered 1% ThS in water for 1 h, and then washed with 70% ethanol, water, and PBS. To co-stain with immunohistochemical staining, the sections were then incubated in blocking buffer and antibody dilution buffer with corresponding antibodies, as mentioned below.

### 4.6. Immunohistochemistry

Immunohistochemistry was performed as described previously [16]. Briefly, sections were blocked in phosphate buffered saline (PBS) containing 1% bovine serum albumin (BSA), 3% normal donkey serum and 0.3% Triton X-100 for 1 h. Then, incubated in PBS containing 1% BSA, 1% normal donkey serum, 0.3% Triton X-100, and primary antibodies, including mouse monoclonal antibodies to Aβ_1–16_ (AB10, Millipore, Burlington MA, USA, MAB5208, 2757889), and glial fibrillary acidic protein (GFAP, Millipore, MAB5804, 1990686); and goat polyclonal antibody to anti-ionized calcium-binding adaptor molecule-1 (Iba-1) antibody (Abcam, Cambridge, UK, ab5076, GR268568-3) overnight at 4 °C. Sections were then incubated in antibody dilution buffer containing Hoechst33258 (Invitrogen, Carlsbad, CA, USA, 2 μg·mL^−1^), Fluorescein isothiocyanate- or rhodamine red X (RRX)-conjugated donkey anti-mouse IgG, RRX-conjugated donkey anti-rabbit IgG or Alexa Fluor 647-conjugated donkey anti-goat IgG (Jackson ImmunoResearch, West Grove, PA, USA, 705-605-147) at room temperature for 2 h. After washed with PBS containing 0.01% Triton X-100, sections were mounted with Aqua Poly/Mount (Polyscience Inc., Warrington, PA, USA) for microscopic analysis using a Zeiss LSM 780 confocal microscopy (Jena, Germany). Representative confocal images were a 10-μm depth with maximal projection. Quantification of amyloid plaque was performed using ImageJ software (https://imagej.nih.gov/ij/). Amyloid plaque burden was calculated by the ratio of AB10-reactive or ThS-positive area to the total area.

Neurogenesis were detected by incubating tissue sections in sodium citrate buffer (10 mM, pH 6.0, 80 °C) for 30 min and 2 M HCl at 37 °C for 30 min, and then in blocking buffer and antibody buffer with primary antibodies and corresponding secondary antibodies, as mentioned above. Primary antibody include mouse anti-BrdU antibody (Santa Cruz, CA, USA, sc-32323) and rabbit anti-doublecortin (DCX) antibody (Abcam, ab18723, GR140153-1). BrdU- or DCX-positive cell number in the subgranular zone (SGZ) was quantified and the linear density of BrdU- or DCX-positive cells per millimeter of SGZ was calculated. The dendritic complexity of newborn neurons in DG was analyzed by laminar quantification of disjointed dendrites [53]. The number of DCX-positive cell bodies (a), primary (b), secondary (c), and tertiary (d) dendrites were counted. The level of primary dendrite sprouting, secondary and tertiary dendritic branches were represented in b/a, c/b, d/c, respectively.

### 4.7. Aβ Enzyme-Linked Immunosorbent Assay (ELISA)

The level of Aβ was determined as described previously [16]. Briefly, Two-step sequential extraction of the brain Aβ using 2% sodium dodecyl sulfate (SDS) and 70% formic acid was processed as described previously [53]. Briefly, homogenate of cortex was mixed with equal volume of homogenization buffer containing 4% SDS and protease inhibitors, sonicated, and then centrifuged at 100,000× *g* for 1 h at 4 °C. The pellet (defined as SDS-insoluble fraction) was re-suspended in 70% formic acid, and then centrifuged at 100,000× *g* for 1 h at 4 °C. The supernatant (defined as SDS-soluble fraction) was collected and neutralized with Tris buffer (1 M, pH 11). Both fractions were stored at −80 °C until ELISA analysis. Aβ level was measured by a human Aβ_1−40_ and Aβ_1–42_ ELISA kit (Invitrogen, KHB3482 and KHB3442). The detailed experiments were performed according to the manufacturer’s protocol.

### 4.8. Immunoblots

Immunoblots was performed as described previously [16]. Briefly, for Western blot analysis, samples (30 μg protein) were separated by SDS-polyacrylamide gel electrophoresis (PAGE) and were then transferred to polyvinylidene difluoride (PVDF) membranes. The primary antibodies used were as follows: mouse anti-GFAP antibody (GFAP, Millipore, MAB360, 2041104), mouse anti-APP antibody (Millipore, MAB348, 25030193), rabbit anti-CTF antibody (Millipore, AB5352, 22051154), rabbit anti-neprilysin (NEP) antibody (Millipore, AB5458, LV1423485), rabbit anti-IDE antibody (Millipore, AB9210, 2769024), mouse anti-β-actin antibody (Millipore, MAB, LV1460528) and goat anti-Iba-1 antibody (Abcam, ab5076, GR268568-3). The secondary antibodies were anti-rabbit IgG antibody conjugated with horseradish peroxidase (HRP; Jackson ImmunoResearch, 711-035-152) and anti-mouse IgG antibody conjugated with HRP (Jackson ImmunoResearch, 715-035-151). The immune complexes were detected using enhanced chemiluminescence reagents (GE Healthcare, Chicago, IL, USA). The images were obtained and quantified using a LAS-3000 Image Analyzer (Fujifilm, Tokyo, Japan).

### 4.9. Burrowing Test and Nesting Test

After oral gavage administration for 70 days, mice were assessed for burrowing test as described previously [46], with minor modifications. In brief, a practice run in group cage on day 70, and two individual tests on day 77 and 80 were performed. Mice were housed in new cage with a thin layer of bedding, and then a cylinder with 230 g of the food pellets was put into the cage at 16:00 next day. Finally, the remaining food pellets in cylinder were weighted after 2 h and overnight. The 2 h measurement of burrowed food pellets in the second individual test was exhibited in the results.

One day after burrowing test, nesting test was performed as described previously [47]. In brief, two Nestlets (5 g) were placed into cage at 1 h before dark cycle, and then the nest score and the weight of unshredded Nestlets were determined after overnight. Nest construction was scored using a six-graded scale [54]. A score of 0 indicates undisturbed Nestlet; 1, Nestlet was disturbed, but nesting material has not been gathered to a nest site in the cage; 2, a flat nest; 3, a cup nest; 4, an incomplete dome and 5, a complete and enclosed dome.

### 4.10. Morris Water Maze Test

After 90-day treatment, spatial memory performance was evaluated using a Morris water maze (MWM) test as described previously [55,56], with minor modifications. In briefly, the water maze apparatus consisted of a circular pool 120 cm in diameter, 40 cm deep, filled to the height of 20 cm with water (temperature 22–24 °C) to cover a platform (diameter 10 cm). The platform was submerged 1 cm below the surface of the opaque water by adding non-toxic white paint. For descriptive data collection, four equal quadrants of the pool was conceptually divided. An computerized video imaging analysis system (Ethovision, Noldus Information Technology Inc., Leesburg, VA, USA) was used to record the swimming paths of black mice in the white background of the maze.

Spatial memory test was conducted to study the spatial memory performance of the mice. All mice were trained in the MWM for 6 days. The platform was always placed in the center of the southwest quadrant. Each mouse was trained to find the platform with four trials a day with an inter-trial interval of 20 min. On each trial, the mouse was lowered gently into the water facing the pool wall at one of the three fixed locations according to a semi-random schedule. In case the mouse did not succeed within 60 s, it was aided onto the platform. At the conclusion of each trial, the mice were allowed to remain on the platform for 30 s whether it had found the platform or not. The escape latency to find the platform were measured in each trial and averaged over four trials.

A spatial probe test was performed wherein the extent of memory was assessed [57]. The time spent in the target quadrant represented the degree of memory that had been obtained after learning during the training period. The 90-s probe trial (one trial without the platform) was assessed on the next day after 6-day acquisition training. The mouse was placed into the pool from the start location at the quadrant opposite of the former platform quadrant. The number of times the mouse crossed the former location of the platform and the time spent in the former platform quadrant were recorded for 90 s. The percentage of time spent in the center *vs.* periphery zones in the probe trial version of the MWM examined. The periphery zone is defined as the area between wall and the circle apart by 10-cm from the wall [51].

### 4.11. Statistical Analysis

The results are expressed as the mean ± standard error of the mean (SEM) and processed for statistical analysis using GraphPad Prism 5 software (La Jolla, CA 92037 USA). The parametric data were analyzed by unpaired two-tailed Student’s *t* test or one-way analysis of variance (ANOVA) with post-hoc Bonferroni’s multiple comparisons tests. The nonparametric data, including crossing times of the platform in MWM test, amount of food pellets in burrowing test and nest score in nesting test, were analyzed using the Kruskal-Wallis ANOVA followed by post hoc Dunnett's multiple comparisons test.

## 5. Conclusions

Taken together, these results suggest that HE-A and HE-S reduce cortical and hippocampal amyloid plaque growth and promoting hippocampal neurogenesis, putatively by inhibiting glial cells and increasing IDE expression in APP/PS1 mice. However, only HE-A was able to decrease Aβ production and the initiation of plaque formation. We also show that HE-A recovers behavioral deficits in APP/PS1 mice. These findings raise the possibility that HE-A may have therapeutic potential for treating AD.

## Abbreviations

Aβ	amyloid-β
AB10-P	AB10 antibody-stained plaque
AD	Alzheimer’s disease
ADL	activity of daily living
ANOVA	analysis of variant
AICD	APP intracellular domain
APP	amyloid precursor protein
sAPPα	soluble APPα
APP/PS1	APPswe/PS1ΔE9
BrdU	5-bromo-2’-deoxyuridine
CTF	C-terminal fragment
DCX	doublecortin
ELISA	enzyme-linked immunosorbent assay
GFAP	glial fibrillary acidic protein
HE	erinacine
Iba-1	ionized calcium-binding adaptor molecule-1
IDE	insulin degrading enzyme
MWM	Morris water maze
NEP	neprilysin
NGF	Nerve growth factor
SGZ	subgranular zone
TCM	traditional Chinese medicine
ThS	thipflavin S
ThS-P	ThS stained plaque
WT	wild type
